# Long-Term Effectiveness of a Multi-Strategy Choice Architecture Intervention in Increasing Healthy Food Choices of High-School Students From Online Canteens (Click & Crunch High Schools): Cluster Randomized Controlled Trial

**DOI:** 10.2196/51108

**Published:** 2024-03-19

**Authors:** Tessa Delaney, Jacklyn Jackson, Christophe Lecathelinais, Tara Clinton-McHarg, Hannah Lamont, Sze Lin Yoong, Luke Wolfenden, Rachel Sutherland, Rebecca Wyse

**Affiliations:** 1 School of Medicine and Public Health University of Newcastle Wallsend Australia; 2 Hunter New England Population Health Hunter New England Local Health District Wallsend Australia; 3 Hunter Medical Research Institute New Lambton Heights Australia; 4 Melbourne School of Population and Global Health University of Melbourne Melbourne Australia; 5 Faculty of Health, School of Health and Social Development Global Centre for Preventive Health and Nutrition, Institute for Health Transformation Deakin University Melbourne Australia

**Keywords:** randomized controlled trial, web-based ordering systems, ordering, menu, menus, prompt, prompts, prompting, nudge, nudges, behavior change, behaviour change, digital intervention, lunch, school, menu labelling, behavioral economics, secondary school, meal delivery apps, public health nutrition, meal, meals, nutrition, nutritional, diet, eating, food, schools, student, students, RCT, randomized, controlled trial, controlled trials, purchase, purchasing, canteen, canteens, choice, choices, architecture

## Abstract

**Background:**

School canteens are a recommended setting to influence adolescent nutrition due to their scope to improve student food choices. Online lunch ordering systems (“online canteens”) are increasingly used and represent attractive infrastructure to implement choice architecture interventions that nudge users toward healthier food choices. A recent cluster randomized controlled trial demonstrated the short-term effectiveness (2-month follow-up) of a choice architecture intervention to increase the healthiness of foods purchased by high school students from online canteens. However, there is little evidence regarding the long-term effectiveness of choice architecture interventions targeting adolescent food purchases, particularly those delivered online.

**Objective:**

This study aimed to determine the long-term effectiveness of a multi-strategy choice architecture intervention embedded within online canteen infrastructure in high schools at a 15-month follow-up.

**Methods:**

A cluster randomized controlled trial was undertaken with 1331 students (from 9 high schools) in New South Wales, Australia. Schools were randomized to receive the automated choice architecture intervention (including menu labeling, positioning, feedback, and prompting strategies) or the control (standard online ordering). The foods purchased were classified according to the New South Wales Healthy Canteen strategy as either “everyday,” “occasional,” or “should not be sold.” Primary outcomes were the average proportion of “everyday,” “occasional,” and “should not be sold” items purchased per student. Secondary outcomes were the mean energy, saturated fat, sugar, and sodium content of purchases. Outcomes were assessed using routine data collected by the online canteen.

**Results:**

From baseline to 15-month follow-up, on average, students in the intervention group ordered significantly more “everyday” items (+11.5%, 95% CI 7.3% to 15.6%; *P*<.001), and significantly fewer “occasional” (–5.4%, 95% CI –9.4% to –1.5%; *P*=.007) and “should not be sold” items (–6%, 95% CI –9.1% to –2.9%; *P*<.001), relative to controls. There were no between-group differences over time in the mean energy, saturated fat, sugar, or sodium content of lunch orders.

**Conclusions:**

Given their longer-term effectiveness, choice architecture interventions delivered via online canteens may represent a promising option for policy makers to support healthy eating among high school students.

**Trial Registration:**

Australian Clinical Trials ACTRN12620001338954, https://anzctr.org.au/Trial/Registration/TrialReview.aspx?id=380546 ; Open Science Framework osf.io/h8zfr, https://osf.io/h8zfr/

## Introduction

### Background

Adolescents internationally are prone to having poor quality diets [1-3], which are associated with a higher risk of obesity, poor mental health and well-being, and an increased risk of chronic diseases during adulthood [4]. In particular, data from the most recent national survey of Australian high school children (aged 12-17 years) found that on average 5.7 (SE 0.2) to 6.6 (SE 0.7) serves of discretionary food choices are consumed per day, contributing to 38%-41% of total daily energy intake [5]. Adolescence represents a transitional life stage, which often coincides with increased autonomy regarding food choices and eating behaviors. Healthy eating interventions that can reach the adolescent population during this key stage are required [6], as dietary behaviors during adolescence have been shown to track throughout the life span [7].

High schools are an ideal setting to deliver interventions to improve adolescent nutrition, as they offer ongoing and widespread access to this traditionally hard-to-reach population [8]. Students have also been shown to consume up to 40% of their daily food intake during school hours, and in Australia over 60% of high school students purchase food at least once per week from their school canteen. However, the foods most commonly purchased from this setting are “less healthy,” discretionary foods high in energy, fat, salt, and sugar [9].

Interventions that incorporate choice architecture strategies (eg, provision of information, changing default options, and using incentives) [10] are effective in improving adolescent diet-related outcomes. A recent systematic review found that out of 137 included choice architecture interventions that aimed to modify child or adolescent diet-related outcomes, 74% were effective [10]. Despite this, of the 137 studies, only 9 were conducted in high schools and while 6 of the 9 studies (67%) were shown to be effective, all of the interventions were short in duration (average 10 weeks) and none assessed long-term effectiveness [11].

Online lunch ordering systems (henceforth referred to as “online canteens”), where students select and preorder their lunch using the web or mobile apps, are common in Australian schools [12]. Online canteens represent the optimal infrastructure to implement choice architecture strategies that support students in selecting healthier foods. The research team recently conducted the “Click & Crunch High Schools” cluster randomized controlled trial (RCT). The trial assessed the short-term (2-month) effectiveness of a multi-strategy choice architecture intervention embedded into an online canteen in increasing the relative healthiness of foods purchased at lunch by high school students. At a 2-month follow-up, relative to controls, intervention students purchased significantly more items classified as “everyday” (healthy +5.5%, *P*<.001) and significantly fewer items classified as “should not be sold” (unhealthy –4.4%, *P*<.001) [13]. Although these initial results are promising, evidence suggests that the effects of behavioral interventions can attenuate over time [14,15]. As such, an assessment of the longer-term impact of the intervention on high school students’ lunch purchases is required to better understand how it contributes to long-term behavior change.

Given digital intervention for public health nutrition is still an emerging field, limited studies have been conducted to assess the sustainability of effective interventions. For example, a 2021 umbrella review of 11 systematic reviews of digital interventions to promote healthy eating in children reported that the effectiveness of such interventions in the medium-term and long-term was not well studied [16]. The research targeting adolescents and high school students is sparser still. As this trial [13] was the first to investigate the effectiveness of embedding choice architecture strategies into online canteen ordering systems in high school students, this longer-term follow-up represents a novel contribution to the public health nutrition literature regarding the sustainability of digital health interventions for this underresearched group.

### Objectives

Therefore, this study aims to assess the long-term effectiveness (baseline to 15 months) of the “Click & Crunch High Schools” intervention on increasing the relative healthiness of school canteen lunch purchases by high school students.

## Methods

### Overview

A description of the trial methods has been previously published [13]. The original trial methods and 2-month follow-up were prospectively deposited on the Open Science Framework on October 23, 2020 [17]. The 15-month follow-up was not preregistered however it was conducted per procedures and outcomes as previously registered.

### Study Design

This cohort study was conducted as a parallel-group, cluster RCT. Consenting high schools that were using an existing online canteen hosted by Flexischools (InLoop Pty Ltd; a commercial online canteen provider and partner on this research) and located in NSW Australia were randomized to receive either a multi-strategy choice architecture intervention delivered via the online canteen infrastructure or a usual practice control (ie, standard online canteen). Outcome data were collected over 8 weeks at baseline (October-December 2020), 2 months (the period immediately following intervention commencement, February-April 2021; results previously published) [13], and again at 15-months postintervention commencement (February-April 2022). This paper reports the 15-month findings.

### Sample and Recruitment

#### Schools

School canteen managers from eligible schools were contacted by mail and telephone to invite study participation. A total of 9 (4 intervention and 5 control) government and nongovernment (ie, independent or catholic) schools located in NSW Australia that enrolled high school students (aged ~12-18 years), and used Flexischools as their online canteen provider were eligible to participate in the 15-month follow-up. Schools were ineligible for the trial if they had participated in another unrelated “online canteen” research program conducted by the team or were a catholic school located within a diocese in which ethical approval had not been obtained.

#### Students

As per prespecified eligibility criteria, students were ineligible for inclusion if; they were in grade 12 at baseline data collection as they were unlikely to be still attending school at the follow-up data collection period; or if they had placed recurring lunch orders set before the intervention period as these orders would not have been exposed to the intervention.

### Randomization and Blinding

Following recruitment, an independent statistician block randomized schools (in blocks of 2 and 4) using a random number function in Microsoft Excel. Randomization was stratified by school sector (eg, government vs nongovernment), as evidence suggests there are differences in the availability of healthy food between the school sectors [18]. Schools were unable to be blinded to their group allocation. However, the intervention was applied centrally, and only students at intervention schools could access the intervention strategies via the online ordering system. All student purchasing data was centrally collected by the online provider, reducing any risk of intervention contamination between the groups.

### Intervention

#### Overview

**Figure 1:**
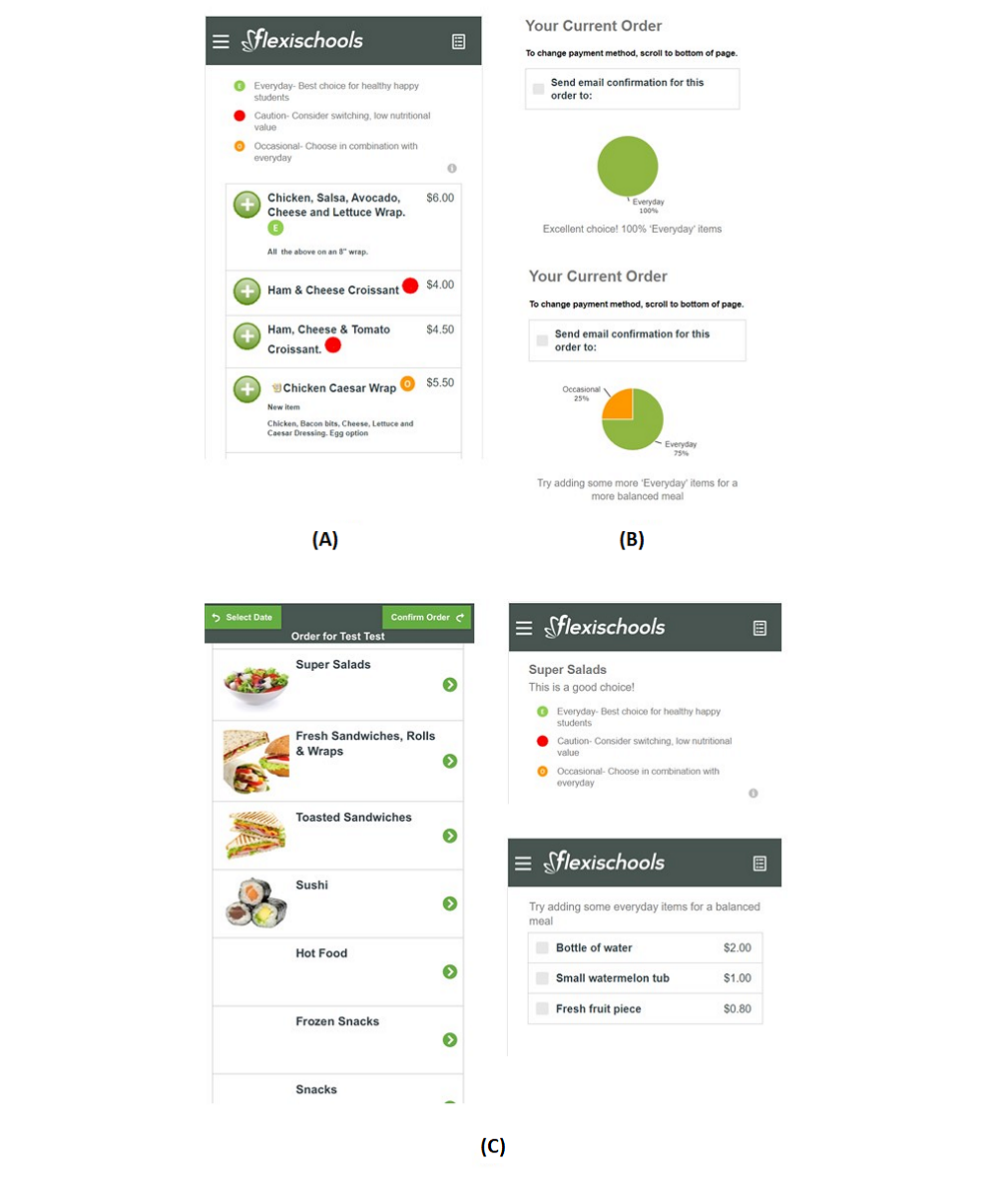
Screenshots from the online canteen showing the following intervention strategies: (A) menu labeling and positioning, (B) feedback, and (C) prompts.

The “Click & Crunch High Schools” intervention is described in full elsewhere [13]. The intervention was underpinned by the principals of choice architecture and sought to encourage the purchase of healthier (ie, “everyday”) items from the school’s online canteen menu. All intervention strategies were integrated into the schools’ existing online canteen and were displayed to students at the point of purchase. All student users of the online canteen at eligible high schools had access to the intervention strategies. The intervention was in place for approximately 15 months (February 2021 to April 2022) until after the 15-month follow-up data collection period. Intervention strategies (see Figure 1) are described in the following sections.

#### Menu Labeling

All menu items were classified as either “everyday,” “occasional,” or “should not be sold” based on the criteria outlined in the NSW Healthy School Canteen Strategy. Menu items were labeled with a small colored symbol: a green circle was added next to “everyday” foods, an amber circle was added next to “occasional” foods, and a red circle was added next to “should not be sold” (“caution”) foods. A “menu label key” appeared at the top of the page (eg, “Everyday- best choice for healthy happy students”; “Occasional- choose in combination with Everyday”; “Caution- consider switching, low nutritional value”).

#### Positioning

“Everyday” menu items and healthier food categories (eg, fruit, salad, and sandwiches) were positioned prominently (ie, first) in the online menu. Research suggests items placed in the middle of menu lists are two times less likely to be purchased than those at the beginning or end [19]. Therefore, the least healthy “should not be sold” items were placed in the middle and “occasional” items were placed last in menu category lists, respectively. Further, “occasional” or “should not be sold” items with multiple flavors (eg, potato crisps) required the user to first “click” on the item before the full list of flavors appeared (eg, plain, salt and vinegar, and chicken).

#### Feedback

Before each lunch order was finalized within the online ordering system, users were shown a personalized summary of the healthiness of their lunch order. The summary included a pie graph displaying the proportion of items in their order that was “everyday,” “occasional,” and “should not be sold,” and a tailored message based on the proportion of “everyday” items in the order (eg, if <99% of items were “everyday”: “Try adding some ‘Everyday’ items for a more balanced meal.” If 100% of items were “everyday”: “Excellent choice! 100% ‘Everyday’ items”).

#### Prompts

When “occasional” or “should not be sold” hot food items were chosen they included a prompt to add a fruit or vegetable snack and water. Healthier menu categories (eg, fruit, salad, and sandwiches) included an appealing image and positive purchase prompt (eg, “This is a good choice”).

To support canteen managers’ understanding of the NSW Healthy School Canteen classification system which underpinned the menu labeling, each canteen manager in the intervention group received a “menu feedback report.” The report included feedback comparing the online canteen menu to the recommendations of the NSW Healthy School Canteen Strategy and provided suggestions on how to improve the relative availability of “everyday” items on the menu.

### Intervention Fidelity

Once every term during the intervention period (approximately every 10 weeks), a member of the research team monitored each school’s online canteen menu via the Flexischools website. They checked that all menu items, including any new items, were correctly classified according to the NSW Healthy School Canteen Strategy, and that the intervention strategies were applied accordingly. If any menu items were found to be unlabeled or incorrectly labeled, the research team would notify Flexischools and provide instructions for how to apply the intervention strategy correctly.

### Control

Control schools did not receive any of the intervention strategies, and were only provided access to the standard online ordering system.

### Data Collection and Outcomes

#### Overview

Student purchasing data were automatically collected and stored by Flexischools. Data were collected over 3 distinct 8-week periods, with baseline occurring from October to December 2020 and long-term follow-up occurring 15 months after the intervention commenced (February-April 2022). The 2-month follow-up was the primary trial end point (data collected immediately following intervention commencement, February-April 2021), and has been previously published [13].

#### Primary Trial Outcomes

The primary trial outcomes at the 15-month follow-up were identical to those at the 2-month follow-up and included the mean percentage of all online lunch items purchased per student that were classified according to the NSW Healthy School Canteen Strategy as (1) “everyday,” (2) “occasional,” and (3) “should not be sold.” The NSW Healthy Canteen Strategy classifies foods as “everyday” based on their alignment with the core foods groups within the Australian Dietary Guidelines (eg, fruit, vegetables, dairy and alternatives, lean meat and alternatives, and grains) [20]. Menu items classified as “occasional” or “should not be sold” are considered “noncore” or discretionary foods that are mostly high in energy, saturated fat, sugar, and salt. Further information on the NSW Healthy School Canteen strategy including the nutrition criteria underpinning the strategy are reported elsewhere [20].

Each canteen menu item was classified against the strategy by a research dietitian using detailed item information (ie, brand, product name, service size, flavor, or recipe) obtained from the canteen manager via telephone or email. Following this, a statistician was able to apply the menu item classification (eg, “everyday”) to the automatically collected purchase data supplied by Flexischools (eg, fresh fruit equaled “everyday”).

#### Secondary Trial Outcomes

##### Energy, Saturated Fat, Sugar, and Sodium Content of Online Lunch Orders

Secondary outcomes included the mean total energy (kJ), saturated fat (g), sugar (g), and sodium (mg) content of online lunch orders. Using previously established procedures, the dietitian generated the nutrition profile for each menu item by using data from food product databases (for commercially packaged menu items [13,21,22]) or FoodWorks (version 9; Xyris Software; for menu items requiring a recipe). The statistician then applied the nutritional profile of each menu item to the student purchasing data provided by Flexischools.

##### Weekly Canteen Revenue From Online Orders

Purchasing data that were automatically collected by Flexischools were used to calculate the mean weekly revenue from all student online lunch orders for the weeks that the canteen was operational at baseline and long-term follow-up. This outcome was assessed to explore any potential adverse effect of the intervention (eg, a reduction in canteen revenue due to the application of the intervention strategies).

#### Other Data

##### School Characteristics

At baseline, school characteristics including the number of student enrollments, year range, sector (eg, government vs nongovernment), school type (combined primary and high school students’ vs high school only), and postcode were obtained from the government “MySchool” website. As the number of high school student enrollments for combined schools was not available on the “MySchool” website, this data was collected directly from the school.

##### Canteen Characteristics

Canteen characteristics including operating days per week, frequency of use, and student grade data were obtained from the student purchasing data supplied by Flexischools.

##### Menu Composition (Pre-Post Intervention)

Using the methods outlined above, a research dietitian assessed the proportion of items on each school’s online menus that were classified as “everyday,” “occasional,” and “should not be sold.” This is reported by intervention and control groups at baseline and 15-month follow-up.

### Statistical Analysis

#### Overview

All outcome data were analyzed in SAS (version 9.3; SAS Institute) under an intention-to-treat (ITT) approach whereby all student lunch orders and schools were analyzed based on the groups they were originally allocated. All nutrition outcomes included data from the student cohort (grades 7-11) that had placed at least one order during the baseline period.

Primary and secondary outcomes were assessed using separate linear mixed models by comparing differences between intervention and control groups over time (baseline to 15 months) through the inclusion of a group-by-time interaction fixed effect. All models included a random intercept for schools (to account for potential school-level clustering), a nested random intercept and random time effect for students (to account for repeated measurements between time points), and fixed effects for the school sector and SEIFA (Socio-Economic Indexes for Australia). All available data (baseline, 2 months, and 15 months) were incorporated into the model.

Consistent with previous publications, the denominator for the unit of analysis for primary trial outcomes was the total number of individual items purchased for each student over the three 8-week data collection periods (baseline: October-December 2020; 2 months: February-April 2021; 15 months: February-April 2022).

Differences in the average weekly revenue (a school-level outcome) were assessed using linear mixed models and included data from all students who had placed any order during any of the data collection periods. School and canteen characteristics were previously reported in the 2-month outcome paper and are included here for context.

Given no differences were observed by subgroups (student grade, frequency of canteen use, or school sector) at the primary trial end point (2 months), no subgroup analyses were conducted at the 15-month follow-up.

#### Sample Size

No sample size calculation was performed for long-term follow-up, sample size estimates were calculated a priori based on the primary trial end point of 2 months [13]. The original sample size required the participation of 10 schools (222 students per school) to ensure a mean detectable difference of 13% of everyday items with 80% power, an intraclass correlation coefficient of 0.05, and an α of .05 at 2-month follow-up.

### Ethical Considerations

The ethical approval for the conduct of this study was provided by the Human Research Ethics Committee of the University of Newcastle (H-2017-0402), and State Education Research Approval Process (SERAP 2018065), as well as relevant Catholic School Dioceses.

## Results

### Overview

**Figure 2:**
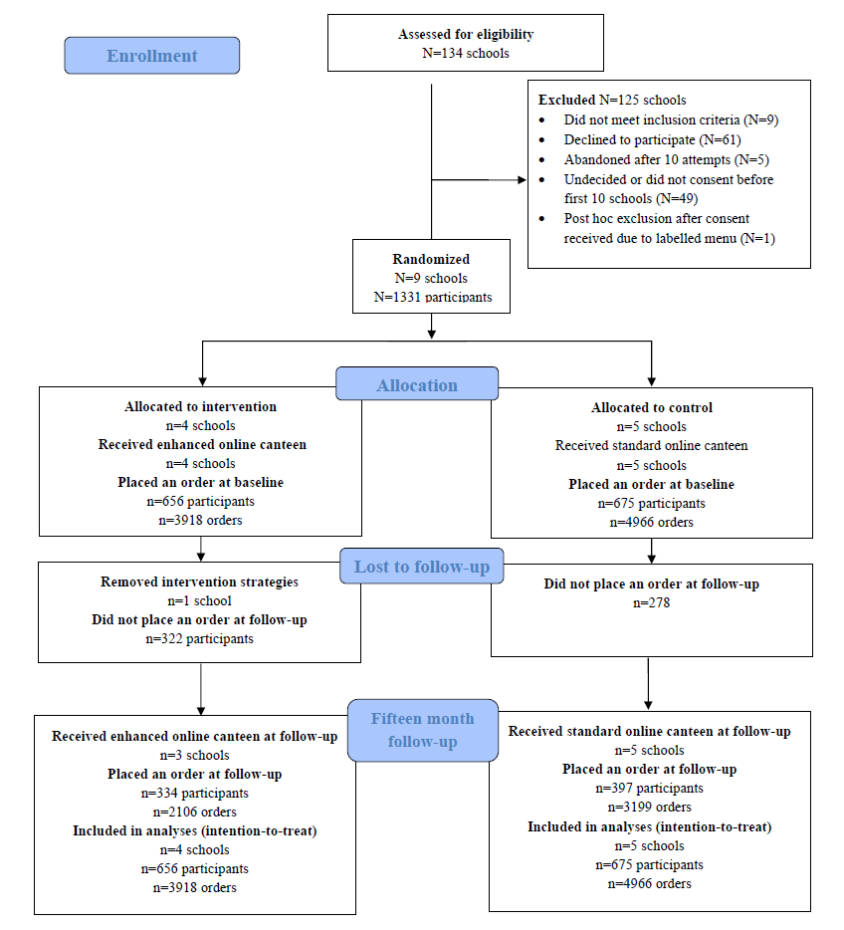
CONSORT (Consolidated Standards of Reporting Trials) diagram.

The baseline characteristics of the sample are presented in Table 1. At baseline, on average, control schools had higher student enrollments compared with intervention schools (mean enrollments 800, SD 318 vs 496, SD 226). All other baseline characteristics were similar between groups (no significance testing was performed). For example, all school canteens operated 5 days per week, the majority of schools were located in areas of most socioeconomic advantage, and the majority of students were in grades 7 to 9. The number of participants and orders at baseline and 15-month follow-up can be seen in Figure 2. While 1331 students from 9 schools provided data at baseline, 332 (25%) students did not place an online order at the 2-month follow-up, and an additional 268 (20%) students did not place an online order at the 15-month follow-up. Of these 268 students, 70 had completed high school (ie, students that were in grade 11 at baseline were no longer at school 15 months later). There were no statistically significant differences between intervention and control participants being lost to follow-up (*P*=.08).

**Table 1:** Characteristics of participating NSW^a^ schools (N=9) and students (N=1331) at baseline.

			Intervention	Control
**School and canteen characteristics (intervention: n=4 schools; control: n=5 schools)**				
	**School sector, n (%)**			
		Government	1 (25)	2 (40)
		Nongovernment^b^	3 (75)	3 (60)
	**School type, n (%)**			
		Combined school (students aged 5-19 years)	3 (75)	2 (40)
		High school (students aged 12-19 years)	1 (25)	3 (60)
	Number of enrollments^c^, mean (SD)		496 (226)	800 (318)
	**Socioeconomic status of school^d^, n (%)**			
		Least advantaged	1 (25)	2 (40)
		Most advantaged	3 (75)	3 (60)
	**Canteen days of operation^e^, n (%)**			
		Five days a week	4 (100)	5 (100)
	**Canteen menu characteristics^f^, n (%)**			
		In total, ≥75% “Everyday” items on menu	2 (50)	2 (40)
		No “Should not be sold” items on menu	0 (0)	0 (0)
	Number of weekly online lunch orders per school^e^, mean (SD)		141 (62)	135 (77)
**User characteristics^e^ (intervention: n=656 participants; control: n=675 participants)**				
	**Grade of student at baseline, n (%)**			
		Grade 7-9	503 (77)	541 (80)
		Grade 10-11	153 (23)	134 (20)
	**Frequency of use, n (%)**			
		High users (≥1 order per week on average)	166 (25)	219 (32)
		Low users (<1 order per week on average)	490 (75)	456 (68)

^a^NSW: New South Wales.

^b^Nongovernment schools were Catholic and independent schools.

^c^Based on publicly available school statistics (MySchool 2020) or verbally from schools (combined schools only).

^d^Socio-Economic Indexes for Australia 2016, based on the postcode of the school locality and dichotomized at the NSW median.

^e^Based on Flexischools purchasing data.

^f^As classified by a dietitian according to the New South Wales Healthy School Canteen Strategy.

### Primary Outcomes

The primary outcomes were the average proportion, per student, of “everyday,” “occasional,” and “should not be sold” online lunch items purchased. Relative to controls, over time from baseline to 15-month follow-up, students in the intervention group ordered on average significantly more “everyday” items (+11.5%, 95% CI 7.3% to 15.6%; *P*<.001), and significantly fewer “occasional” (–5.4%, 95% CI –9.4% to –1.5%; *P*=.007) and “should not be sold” items (–6%, 95% CI –9.1% to –2.9%; *P*<.001) in an intention-to-treat (ITT) analysis (Table 2).

**Table 2:** Differences in intervention and control groups over time for primary and secondary outcomes^a^.

			Intervention				Control				Intervention versus control^a^		
			Baseline (n=656), mean (SD)		15-Month follow-up (n=334), mean (SD)		Baseline (n=675), mean (SD)		15-Month follow-up (n=397), mean (SD)		Main analysis (15-month versus baseline)		
											Group by time differential effect (95% CI)		*P* value
**Primary outcomes**													
	Percentage per student of lunch items that are “everyday”	44.3 (34.3)		51.0 (34.0)		43.2 (36.3)		39.4 (36.0)		11.5 (7.3 to 15.6)		<.001^b^	
	Percentage per student of lunch items that are “occasional”	30.7 (30.8)		30.4 (29.7)		40.9 (35.7)		45.7 (37.7)		–5.4 (–9.4 to –1.5)		.007^b^	
	Percentage per student of lunch items that are “should not be sold”	25.0 (30.9)		18.6 (28.3)		16.0 (24.7)		15.0 (23.2)		–6.0 (–9.1 to –2.9)		<.001^b^	
**Secondary outcomes**													
	Energy (kJ) per student lunch order	2172.5 (976.9)		2211.7 (922.3)		1992.8 (793.1)		1943.4 (796.6)		48.8 (–34.6 to 132.2)		.25	
	Saturated fat (g) per student lunch order	7.3 (5.7)		7.0 (5.0)		5.7 (4.4)		5.5 (4.4)		–0.0 (–0.5 to 0.5)		.99	
	Sugar (g) per student lunch order	22.4 (22.8)		22.5 (21.0)		15.3 (16.2)		12.8 (15.0)		1.7 (–0.1 to 3.5)		.07	
	Sodium (mg) per student lunch order	778.9 (354.1)		795.9 (368.8)		808.3 (398.2)		789.3 (384.1)		0.35 (–36.2 to 36.9)		.99	
	Weekly revenue (Aus $)^c^ per school	896.1 (449.9)		1243.8 (485.9)		769.6 (372.8)		1798.5 (653.9)		–673.4 (–1252.6 to –94.2)		.03^b^	

^a^All models included a random intercept for school, a nested random intercept and random time effect for students, and fixed effects for the school sector and Socio-Economic Indexes for Australia. All available data were incorporated into the model (baseline, 2-months, and 15-months) to describe purchasing patterns over time.

^b^*P*<.05.

^c^All $ amounts are in Aus $. A currency exchange rate of Aus $1 = US $0.65 was applicable as of February 2024.

### Secondary Outcomes

#### Average Energy, Saturated Fat, Sugar, and Sodium Content of Online Lunch Orders

There were no between-group differences over time (baseline to 15-month follow-up) in the average energy (+48.8 kJ, 95% CI –34.6 to 132.2; *P*=.25), saturated fat (–0.0 g, 95% CI –0.5 to 0.5; *P*=.99), sugar (+1.7 g, 95% CI –0.1 to 3.5; *P*=.07), or sodium (+0.35, 95% CI –36.2 to 36.9; *P*=.99) content of student lunch orders.

#### Weekly Online Canteen Revenue (Potential Adverse Effect)

While both intervention and control groups increased in revenue (a currency exchange rate of Aus $1=US $0.65 applies) over time (intervention-group baseline: Aus $896.10; intervention-group 15-month follow-up: Aus $1243.80; control-group baseline: Aus $769.60; control-group 15-month follow-up: Aus $1798.50), the increase in the intervention group was significantly lower than the increase in the control group (differential effect –Aus $673.40, 95% CI –Aus $1252.60 to –Aus $94.20; *P*=.03). To further qualify this effect, a post hoc exploratory analysis was undertaken to explore if students spent more money per order between intervention and control groups over time. The exploratory analysis found no difference in the average spend per student order by intervention and control groups over time (difference Aus $0.07, 95% CI –Aus $0.14 to Aus $0.28; *P*=.48).

### Other Data

#### Menu Composition

While no significance testing was performed, the proportion of “everyday,” “occasional,” and “should not be sold” items available on menus at baseline and 15-month follow-up were similar for the intervention and control schools (Table 3).

**Table 3:** Menu composition at baseline and 15-month follow-up.

Menu item classification	Intervention		Control	
	Baseline availability, mean %	15-month follow-up availability, mean %	Baseline availability, mean %	15-month follow-up availability, mean %
Everyday	69.4	69.7	68	69.1
Occasional	14.9	15.5	20	21.7
Should not be sold	15.7	14.8	12	9.2

#### Intervention Fidelity

Of the 4 intervention schools, 3 had 99% (1256/1269 items) of their menu labeled correctly during the 15-month intervention period. The remaining school removed all of their labels in the last 12 weeks of the intervention, resulting in 81% fidelity across the 15-month intervention period.

## Discussion

### Principal Results

This is the first study to assess the long-term effectiveness of an intervention embedded within an online lunch ordering system for high school students and is one of few studies to assess the long-term effectiveness of food choice architecture interventions more broadly [10,23,24]. The Click & Crunch High Schools cluster RCT found that intervention students, relative to control, ordered significantly more healthy “everyday” items and significantly fewer “less healthy” items from baseline to 15-month follow-up. There were no between-group differences over time in the average energy, saturated fat, sugar, and sodium content of high school student online lunch orders. This study found that the online canteen revenue for both groups increased over 15 months, however, the revenue in the intervention group grew more slowly than the control group. These findings were surprising, given other trials in the school food setting have found no differences between groups in revenue [25-27].

### Comparison With Prior Work

While there is limited research to draw direct comparisons of this study, systematic reviews of the school setting have found that very few studies have assessed the long-term effectiveness of nutrition interventions in high schools [24,28]. A systematic review by Mingay and colleagues [24] found that only 6 of 35 studies assessed the long-term effect (≥12 months) of school meal interventions on the selection or purchase of healthier foods by high school students. Similar to our study, the review found mixed evidence for studies that included multiple dietary outcomes (eg, nutrients vs food groups) in their assessment of long-term effectiveness. In contrast to our study, the review found that shorter interventions (<3 months) had a greater effect on dietary outcomes for high school students [24]. Contrary to these review findings, our study found that there was a greater magnitude of effect at 15-months compared to the 2-month follow-up (previously reported) [13]. For example, at 2 months the Click & Crunch High School intervention was effective in increasing “everyday” items (+5.5%, *P*<.001) and decreasing “should not be sold” (–4.4%, *P*<.001) items purchased by students, with no difference in the purchase of “occasional” items (–1.2%, *P*=.47) [13]. At the 15-month follow-up, the magnitude of effect was greater than that observed at 2 months and the decrease in “occasional” items purchased was now significant (15-months: everyday +11.5%, *P*<.001; occasional –5.4%, *P*=.007; should not be sold –6%, *P*<.001). The increase in effect size over time may in part be explained by the high intervention fidelity, the intervention type (choice architecture vs food provision), and the number of strategies employed in this trial. Furthermore, the greater length of time that students were exposed to the intervention may have increased the likelihood of habitual patterns in the purchasing of more healthy foods. The sustained intervention effectiveness may also be attributable to the precommitment involved with “preordering,” which may prevent impulse purchasing of “less healthy” foods due to hunger-based cues [10].

Although this is the first RCT to describe the long-term effectiveness of an online choice architecture intervention in the high school setting (enrolling students aged ~12-18 years) a similar pattern of results has been found in related food service settings [29,30]. For example, a longitudinal study undertaken with adults in a large hospital cafeteria found that a 2-year choice architecture intervention involving traffic light labeling, product placement, and promotion increased the sale of “healthy” items by 5% and decreased the sale of unhealthy items by 3% (*P*<.001). In the primary school setting (aged 5-12 years), the same Click & Crunch intervention was found to be effective at improving healthy food purchases by primary school students at 18 months (+3.8% “everyday” and –2.6% “less healthy” items purchased) [29]. Such findings demonstrate the potential merit of the Click & Crunch intervention on improving the nutritional quality of both primary and high school student online lunch purchases over both the short and longer term and challenge the previously held notion that choice architecture interventions may attenuate over time due to their “novelty effect” or “label fatigue” experienced by end users [10].

### Broader Implications of This Research

The findings of this trial may have broader relevance to the online food ordering systems more generally. The World Health Organization has identified the need to leverage online food delivery systems for public health benefits [31]. This is the first trial to embed public health nutrition strategies within online food ordering systems for adolescents. With the exponential rise in related meal delivery app use particularly by adolescents and young adults (aged >15 years) [32], these research findings are likely to be of interest to policy makers investigating how to leverage such systems for public health benefit.

### Strengths and Limitations

This cluster RCT had several strengths, including the robust trial design, objectively collected purchase data, and the use of a real-world online lunch ordering system to deliver simple choice architecture strategies. Importantly, it is one of few studies assessing the long-term effects on food purchase or consumption of a choice architecture intervention and the first to do so in the high school setting. Despite this, this study had several limitations. In addition to those already discussed in the 2-month follow-up [13], this study did not assess intervention costs or acceptability which are key determinants of intervention scalability [33]. Therefore, to support public health decision-making regarding the scalability of these interventions, future research that explores the acceptability of the intervention to end users (high school canteen managers and students) and intervention costs including cost-effectiveness may be warranted. Furthermore, as this study did not find differences in nutrient outcomes (energy, saturated fat, sugar, and sodium), future research may be required to understand the differential effect of alternate menu labeling systems (eg, kJ labeling) on nutrient-based outcomes. Finally, as outlined in the 2-month follow-up [13], to achieve population-wide improvements in adolescent nutrition this intervention should be considered in addition to broader public health nutrition strategies that reach both users and nonusers of online canteens in the high school setting.

### Conclusions

Despite the limitations, this is the first RCT to explore the long-term effectiveness of a choice architecture intervention embedded within an online canteen targeting high-school students and one of only a few choice architecture interventions delivered in the high-school setting. The findings suggest that there are long-term effects of up to 15 months after intervention commencement, including a significant increase in healthy “everyday” items and a significant reduction in less healthy “occasional” and “should not be sold” items. This provides valuable evidence about the potential long-term effect of choice architecture interventions delivered via online canteens on adolescent school lunch ordering and may be useful to policy makers interested in improving adolescent diet within the high school setting. Further research is required to determine the feasibility of disseminating such interventions to schools at scale, and if these effects transfer to other online food environments targeting different end users (ie, adults and health care workers) such as workplaces, hospital settings, and the fast food sector.

## Appendix

Multimedia Appendix 1 CONSORT eHEALTH checklist.

## Abbreviations

ITT: intention-to-treat
NSW: New South Wales
RCT: randomized controlled trial
SEIFA: Socio-Economic Indexes for Australia

## Data Availability

The data sets generated during or analyzed for this study are available from the corresponding author on reasonable request, pending ethics approval.
